# Prevalence of antibodies against COVID-19 in the staff of a COVID-19 regular ward

**DOI:** 10.3205/dgkh000344

**Published:** 2020-05-14

**Authors:** Igor Alexander Harsch, Marcin Skiba, Peter Christopher Konturek, Jörg Epstude

**Affiliations:** 1 Department of Internal Medicine II, Thuringia Clinic “Georgius Agricola”, Saalfeld/Saale, Germany; 2 Department of Hospital Hygiene, Thuringia Clinic “Georgius Agricola”, Saalfeld/Saale, Germany

**Keywords:** Antibodies, COVID-19, SARS-CoV-2, Serology

## Letter

Dear editor,

The SARS-Cov-2 infection has spread worldwide. The clinical spectrum of the infection is highly variable. It can range from courses that essentially go unnoticed by the person infected up to the most serious pulmonary infections with fatal consequences [1].

The extent of infections in the population that have been run their course or were almost symptom-free is currently a research priority. It may lead to more precise prediction models for the pandemic’s further development. Recently, among 957 employees of the University Clinic of Münster, 5.4% tested positive in swab testing [2].

The particular focus of our in-house investigation was to collect data on how many permanent medical and nursing staff members of the CORONA-19 regular ward could have already experienced such an infection. This has recently been made possible by antibody identification in the serum [3]. To our knowledge, data in such a subgroup have not yet been published.

After obtaining informed consent and with approval by the Ethics Committee of the State Medical Association of Thuringia, we examined the antibody titers of the two doctors, 15 nurses and one housekeeper (n=18) assigned to the ward from April 16 to 21 2020. The mean age was 44.9 yrs, range 21–60 yrs.

For antibody determination against SARS-CoV-2, we used an ELISA (EUROIMMUN™, a PerkinElmer, Inc. company) which is CE (Conformité Européenne)-certified and IVD (In Vitro Diagnostic)-approved, that was additionally validated in-house by our lab. The specifity of the test for IgG is specified as 98.5% by the distributor and as 92.5% for IgA. The same assay was used in the COVID-19 case-cluster study by Streeck et al. [unpublished data] during their investigations in the Heinsberg district in North Rhine-Westphalia [one of the early “hot spots” in Germany].

In terms of our ward, IgG antibodies against COVID-19 (IgG formation usually 10-14 days after confirmed symptom onset) were not detected in any staff member, nor were IgA antibodies (“early” antibodies). Although the validity of the assay is not yet undisputed, we observed elevated IgG titers in persons with confirmed SARS-CoV-2 pneumonia in March from our clinic (data not shown).

There is no common strategy for the surveillance of staff members of wards dedicated to the isolation and therapy of patients with suspected or confirmed COVID-19 infections. Since March 19, a weekly oropharyngeal smear has been mandatory in our clinic for all staff of this ward, with negative results to date. With this in mind, it was not surprising that we did not detect elevated IgG or IgA antibodies.

The results match the low prevalence of COVID-19 infection in the region and also speak for the effectiveness of the usual protective measures for the staff: The German Federal State of Thuringia (population: 2.1 million) has a relatively low number of confirmed COVID-19 infections. As of April 21, 1798 cases had been reported by the Robert Koch Institute [4]. On the other hand, the first patient with confirmed SARS-CoV-2 pneumonia in Thuringia was treated in this very ward (March 2 until March 17 2020), followed by 17 patients to date with confirmed COVID-19 infection (and six times the number of unconfirmed cases that were thus transferred into other wards of the clinic after testing negative).

Even if the number of cases in this sample is small, the data does not indicate a high degree of past COVID-19 infections in the population of this region thus far.

Our observations clearly show the importance not only of implementing but also maintaining hygienic standards. However, despite the current restrictions in everyday life, the risk for the staff to become infected in the social or home environment outside the clinic remains unaffected.

## Notes

### Competing interests

The authors declare that they have no competing interests.
